# Combined Effects of Deficit Irrigation and Biostimulation on Water Productivity in Table Grapes

**DOI:** 10.3390/plants13233424

**Published:** 2024-12-06

**Authors:** Susana Zapata-García, Abdelmalek Temnani, Pablo Berríos, Laura Marín-Durán, Pedro J. Espinosa, Claudia Monllor, Alejandro Pérez-Pastor

**Affiliations:** 1 Departamento de Ingeniería Agronómica, Universidad Politécnica de Cartagena (UPCT), Paseo Alfonso XIII, 48, 30203 Cartagena, Spain; susana.zapata@upct.es (S.Z.-G.); abdelmalek.temnani@edu.upct.es (A.T.); pablo.berrios@upct.es (P.B.); laura.mduran@edu.upct.es (L.M.-D.); 2 Europe, Middle East & Africa Region (EMEA) Plant Health Portfolio, FMC Agricultural Solutions, 28046 Madrid, Spain; pedro.espinosa@fmc.com; 3 Plant Health Portfolio, FMC Agricultural Solutions, 28046 Madrid, Spain; claudia.monllor@fmc.com

**Keywords:** biostimulant, leaf gas exchange, mycorrhization, precision irrigation, sensorization, soil water content, sustainability, root growth

## Abstract

Biostimulation and precision irrigation are strategies that increase the sustainability of agriculture, and both have been widely studied in table grapes, but their interaction is a new approach for viticulture. The objective of this field trial was to assess the physiological effects of water deficit on table grapes pretreated for two consecutive years with five different biostimulation programs. Therefore, during the first year, vines were preconditioned with biostimulants composed of microorganisms, seaweed, and plant extracts and compared to an untreated control. During the second year, the same biostimulation treatments were evaluated under two different irrigation schedules: (i) farmer irrigation (FI), according to a farmer’s criteria; and (ii) a deficit irrigation program, precision irrigation (PI), in which irrigation water was reduced from the post-veraison period to harvest, setting a threshold for allowable soil water depletion of 10% with respect to field capacity in order to minimize water leaching. The water inputs in the treatments under PI were reduced by 30% with respect to the FI treatment. While the deficit irrigation treatment clearly affected the plant water status indicators, biostimulation enhanced the root colonization by mycorrhizae and showed a trend of increased new root density. The combined effect of biostimulation and PI was shown to be an efficient strategy for optimizing the available resources, promoting the yield precocity.

## 1. Introduction

Viticulture holds a significant importance in Spain, not only due to its cultural and historical meaning but also because of the economic value it generates. Nowadays, Spain possesses the largest cultivated area for vineyards in the world, accounting for 13.8% in 2021 [1]. The Murcia Region, located in the southeast of the country, produced 192,740 tons of table grapes in 2022, making it the leading province in Spain table grape production [2]. Additionally, the region’s exports are relevant, accounting for 67.6% of Spain’s total grape exports in 2022 [3]. To export such a large quantity, it is essential that the product possesses, among others, excellent organoleptic qualities. That is why new varieties, such as “Sweet celebration”, are cultivated nowadays; this seedless variety has bright dark red berries, a crunchy texture, and a sweet taste [4].

To achieve high-quality traits, effective crop management is vital, with irrigation being a key factor. The southeast of Spain is a semiarid region that faces severe challenges due to water scarcity. This problem is being exacerbated by climate change [5], making irrigation water an even more valuable resource for agriculture nowadays. Deficit irrigation has been used for optimizing water use in irrigated agriculture [6]. Linked to that idea, precision irrigation (PI) strategies have been developed with the goal of mitigating the adverse effects of water deficits by reducing the amount of water applied to crops. This can help to increase water productivity and water use efficiency by ensuring that crops receive the right amount of water at the right time. Typically, these strategies include the use of advanced technologies such as sensors and remote sensing data to monitor and manage the water needs of crops more accurately. For instance, they can identify spatial variability in water requirements across a field [7] or enable adjustments to irrigation schedules based on real-time soil moisture data [8]. Thanks to digitalization and the use of soil moisture sensors, farmers have been able to optimize water productivity and nutrient use efficiency [9,10]. Digitalization also facilitates the adoption of new strategies such as precision irrigation. By using real-time sensors to assess irrigation, the leaching of water and nutrients below the root system can be reduced [8], and a better use of available water resources can be made, therefore contributing to achieving the sustainable development goals [11].

Particularly, the management of vineyards faces the challenge of excessive vigorous growth, which can be reduced by the implementation of different deficit irrigation strategies [12,13].

Among the biotechnologies incorporated in agriculture, the use of biostimulants has gained significant attention [14]. These are substances of diverse nature or microorganisms that, when applied to plants or the rhizosphere, stimulate natural processes to enhance the crop yield, nutrient use efficiency, crop quality traits, or plant tolerance to various biotic and abiotic stresses [15]. Biostimulants have been used to mitigate these stresses in viticulture, as recently reviewed by Monteiro et al. [16], who highlighted the potential of biostimulants as an alternative to reduce chemical inputs in agriculture and therefore increase sustainability while maintaining the economic viability of the crop. Several types of biostimulants have demonstrated beneficial effects on the growth and photosynthetic efficiency of grapevine seedlings under high-temperature stress, though they stimulate different levels of response [17]. Additionally, some researchers have assessed the effects of drought conditions on different biostimulants (amino acid, humic acid, fulvic acid, seaweed extract, and protein hydrolysates), concluding that they alleviate the adverse effects of drought stress [18,19].

As du Jardin [20] mentioned in his review, the development of every biostimulant needs to be tested in field situations to assess the beneficial effects. Biostimulant products’ formulation with specific application rates and timing in each particular crop [15,21] need to be analyzed to define their claims [22]. Therefore, in this trial, we analyzed the agronomical and physiological effects of four different biostimulation strategies applied to table grapes over two years and how these treatments affected the hardening of the vines when they faced a water deficit.

## 2. Results

### 2.1. Climatic Condition and Irrigation Water Applied

**Figure 1:**
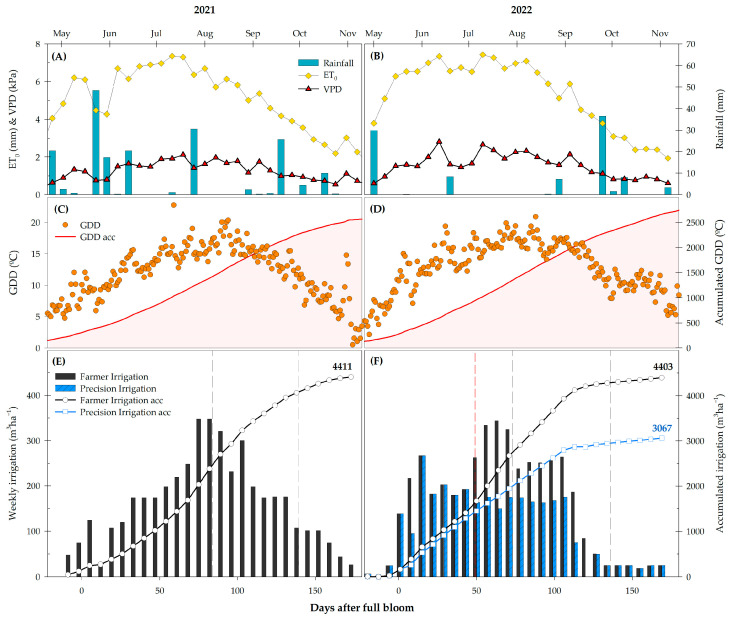
Weekly evolution of the climatic parameters: Vapor pressure deficit (VDP), reference crop evapotranspiration (ET_0_), and rainfall (**A**,**B**). Daily growing degree days (GDDs) and accumulated GDDs from sprouting (−62 DAFBs) (**C**,**D**). Weekly and accumulated irrigation applied for each season 2021 (**E**) and 2022 (**F**). DAFBs: days after full bloom. 0 DAFBs corresponds to 14 May 2021 and 17 May 2022, respectively, for each season. The gray dashed lines indicate the harvest period, and the red dashed line in 2022 indicates the beginning of the irrigation reduction in PI with respect to farmer irrigation.

For both years, the period from June to August coincided with the highest evaporative demand, when the values of vapor pressure deficit (VDP) and reference crop evapotranspiration (ET_0_) reached their maximums, around 2.5 kPa and 7.4 mm day^−1^, respectively (Figure 1A,B). As the temperatures were higher during the second season, the accumulated growing degree days (GDDs) calculated from sprouting (−62 days after full bloom, DAFBs) were increased to 174 GDDs during 2022 (Figure 1C,D). Rainfall was 90.3 mm higher in 2021 than 2022, mainly due to the rainy period at the end of May (Figure 1A,B).

Farmer weekly irrigation varied throughout the season according to the climatic demand, always satisfying the crop needs according to crop evapotranspiration (ET_c_). The volumes applied increased gradually from May, 50 m^3^ ha^−1^ (2021) or 20 m^3^ ha^−1^ (2022), to 350 m^3^ ha^−1^ during July–August. The total water amount applied in the farmer irrigation (FI) treatment was 4411 m^3^ ha^−1^ in 2021 and 4403 m^3^ ha^−1^ in 2022 (Figure 1E,F). In 2022, the irrigation reduction in the precision irrigation (PI) treatment was applied from 49 DAFBs to the end of harvest, saving 1336 m^3^ ha^−1^ a 30.3% reduction compared to the FI treatment at the end of the season (Figure 1F).

### 2.2. Soil Volumetric Water Content

**Figure 2:**
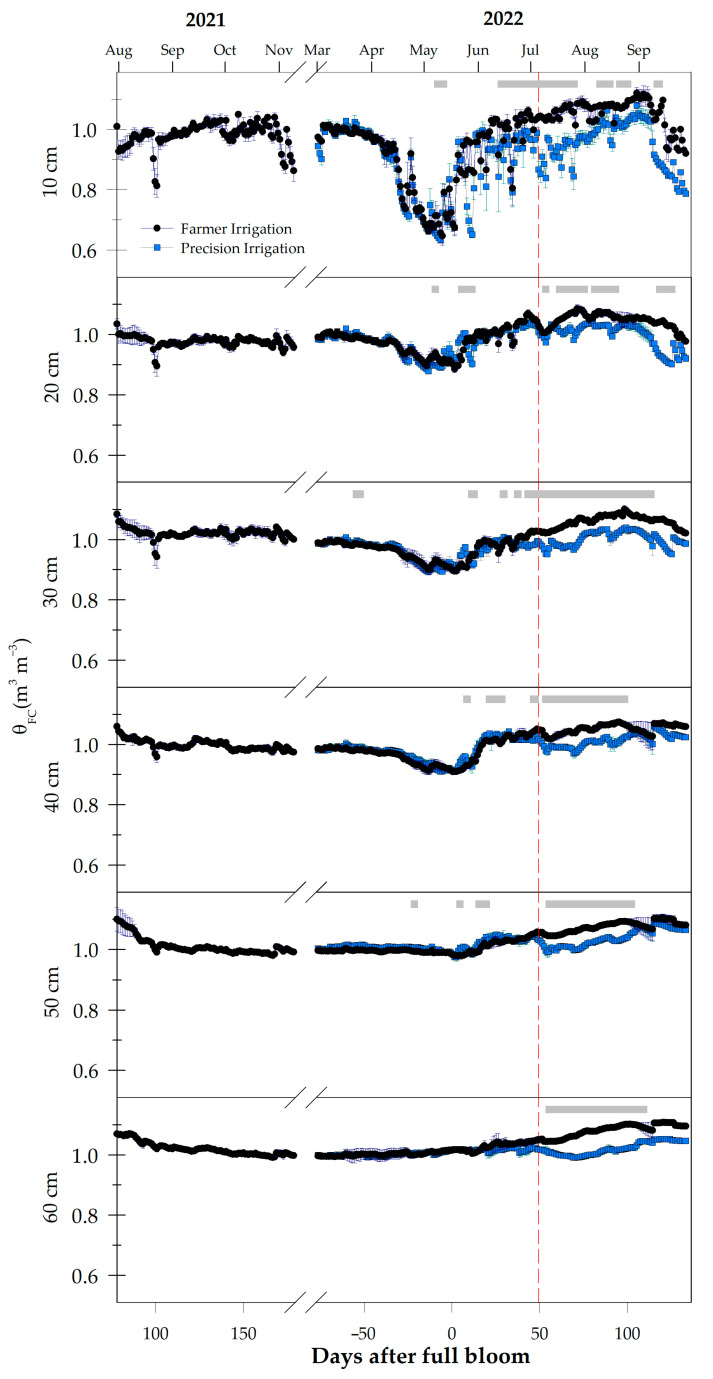
Daily evolution of the minimum volumetric water content in the soil profile (0 to 60 cm depth), relativized to the field capacity for each depth during the experimental period. The red dashed line indicates the beginning of the irrigation reduction in PI. Means ± SE, *n* = 3. The gray squares indicate the days with significant differences between irrigation treatments (*p* ≤ 0.05).

The evolution of the soil volumetric water content, relative to the field capacity at each depth (θ_FC_), is represented in Figure 2. For most of the trial period, FI remained close to field capacity. However, the elevated climatic demand between April and June led to a reduction in the soil volumetric water content at a depth of 10 cm (Figure 2). From that moment onward, the θ_FC_ increased to the saturation point throughout the entire soil profile, from 10 to 60 cm depth, due to the additional water volume applied by the farmer in response to the increased climatic demand. In May 2022, a strong water depletion in the soil was observed. During those weeks, as the VPD and ET_0_ drastically decreased, the crop was not irrigated (Figure 1). The rainwater did not reach the root system because of the plastic net covering the vines; as a consequence, the soil water absorption was increased, drying the soil (Figure 2).

The application of the deficit irrigation in PI, starting on 49 DAFBs 2022, showed significant differences between both irrigation programs at all depths. The most sensitive profile for assessing the different irrigation regimens was at a depth of 30 cm, where the differences were shown during the entire period (Figure 2).

### 2.3. Vines Water Status

In both years during earlier stages such as flowering, the vines showed higher leaf gas exchange values, net photosynthesis (Pn), and stomatal conductance (Lc) compared to after the fruit set (Table S1). In 2021, treatments in which the foliar seaweed extract was applied increased their photosynthetic rate at flowering. In this period, the values were significantly lower in T4 and T5, which corresponded to biostimulated with nonfoliar products and the untreated control, respectively. For the rest of the dates, no differences between treatments were found (Table S1).

The average values for leaf gas exchange parameters for the season of 2021 revealed differences in Pn and Lc, with T1 being the treatment with the highest values in both parameters, while T5 had the lowest (Table 1). Unlike the 2021 season, the vines under the farmer irrigation program in 2022 did not show significant differences between treatments. However, the average values for Pn and Lc increased in 2022 compared to the previous season under the same irrigation program (Table 1). Unlike 2021, in 2022, the biostimulated treatments under FI did not show significant differences in the leaf gas exchange parameters (Pn, Lc) or the stem water potential (Ψ_s_) for either the individual dates measured or for the average value of the season.

The irrigation factor in 2022 caused the Ψ_s_ to decrease in PI from the beginning of its application at 49 DAFBs. Differences in this parameter were observed during the rest of the season, even at 134 DAFBs, when irrigation was recovered. The gas exchange parameters were affected from 98 DAFBs on, with decreased values for Pn and Lc in the treatments under the PI program. On this same date, during the phenological stage of ripening, the biostimulation factor showed differences in leaf conductance, increasing its values under both irrigation programs.

Although the seasons’ average showed that the biostimulation factor did not significantly influence those parameters, the irrigation factor did. The farmer irrigation increased the photosynthetic rate and leaf conductance, promoting a higher stomatal opening.

### 2.4. Root Density

**Figure 3:**
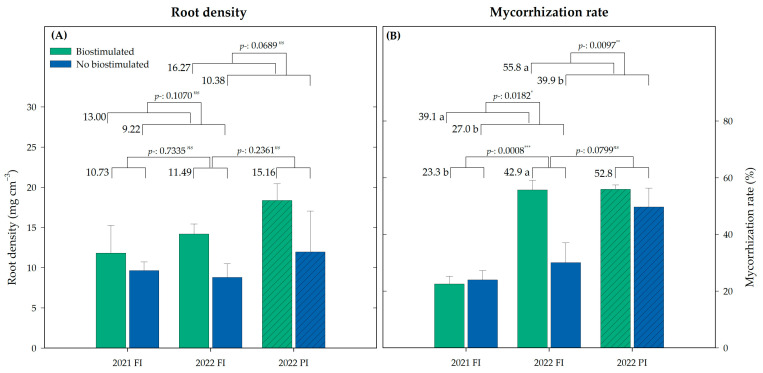
(**A**) Root density and (**B**) mycorrhization rate for biostimulated (T1–T4) and not biostimulated (T5) vines under the different irrigation programs (farmer irrigation (FI) or precision irrigation (PI)) in 2021 and 2022. Bars represent means ± SE (*n* = 4). Different letters indicate significant differences for the factors biostimulation, year, or irrigation in each parameter according to Duncan’s test (*p* < 0.05). Average value for each factor is shown. *: *p* < 0.05; **: *p* < 0.01; ***: *p* < 0.001; *ns*: not significant.

The biostimulation and the different irrigation programs did not show significant differences in the fine root density of the vines for any season (Figure 3A). The root density for the vines under FI remained constant over the two years, with a slight tendency to increase annually.

The nonbiostimulated treatment (T5) showed an increasing trend when irrigation was reduced under the PI program. The biostimulated vines showed a positive trend in increasing the root density over the years, a trend that was even more evident when subjected to PI, averaging 6.5 mg cm^−3^ more than the previous year.

### 2.5. Mycorrhization Rate

After the 2021 growing season, the treatments did not show any effect on the mycorrhization rate, presenting, on average, a root colonization rate of 23% for the whole plot. Contrarywise in 2022, the cumulative effect of biostimulants had an influence on it (Figure 3B). Under the FI program, T4 was the treatment with the highest colonization rate of 64%, and the untreated control (T5) the lowest, with 30%. Under the PI program, the treatments T1 and T4 showed the highest mycorrhization rates of 67 and 61%, respectively, while T2 had the lowest, with 40%.

Even though exogenous mycorrhiza was applied in all plots between the two samplings, the mycorrhization rate only increased in the biostimulated treatments (T1–T4) (Figure 3B). The different irrigation programs carried out in 2022 did not cause significant differences in the root colonization (Figure 3B). However, it should be noted that the nonbiostimulated treatment (T5) showed a nonsignificant trend to increase the mycorrhization rate under precision irrigation (*p* = 0.0635 *^ns^*).

### 2.6. Starch and Soluble Sugar

**Figure 4:**
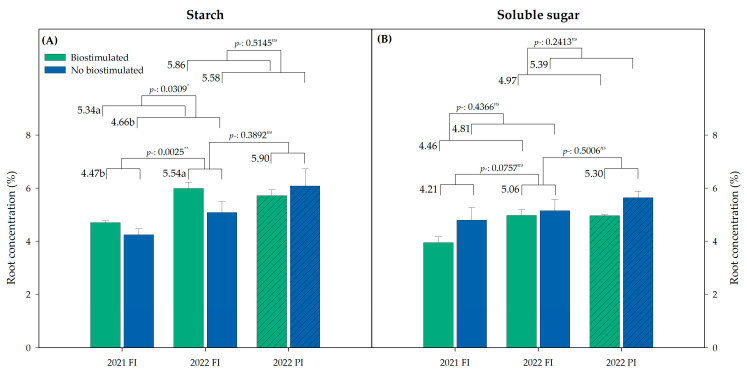
Root starch (**A**) and soluble sugar (**B**) concentration (%, p/p) for biostimulated (T1–T4) and not biostimulated (T5) vines under the different irrigation programs (farmer irrigation (FI) or precision irrigation (PI)) in 2021 and 2022. Bars represent means ± SE (*n* = 4). Different letters indicate significant differences for the factors biostimulation, year, or irrigation in each parameter according to Duncan’s test (*p* < 0.05). Average value for each factor is shown. *: *p* < 0.05; **: *p* < 0.01; *ns*: not significant.

The starch and soluble sugars concentration in the vine roots did not show significant differences between treatments. Starch content was increased in 2022 in all treatments, particularly in those treated with biostimulants (T1–T4), reaching nearly 6% (p/p), and was significantly higher than the nonbiostimulated treatment under FI (Figure 4A). Soluble sugars were unaffected by the year, biostimulation, or irrigation treatments (Figure 4B).

### 2.7. Yield

The total yield did not significantly differ among treatments during the experimental period for any of the conditions evaluated (Table 2), but it varied between seasons. In 2021 and 2022, the farmer irrigation program averaged 41.1 and 37.0 t ha^−1^, respectively, while the precision irrigation was 40.8 t ha^−1^. The sum of the harvests I to III represented, on average, 51% and 58% of the total harvest for FI in 2021 and 2022, respectively. The different biostimulation treatments did not significantly differ from each other, although T2 and T4 showed the highest yield values both years. On the other hand, for all the treatments under PI, harvests I to III represented 88% of the total yield. T3 and T4 had more than 90% of the total harvest in the first three pickings. In general, treatments under PI achieved a significantly higher yield than treatments under FI in the same period (*p*-value < 0.0001), which show a relationship between irrigation reduction and harvest precocity. For the later harvests (IV to VI), the trend was the opposite, with 42% of the yield in FI and 12% in PI (*p*-value < 0.0001).

**Figure 5:**
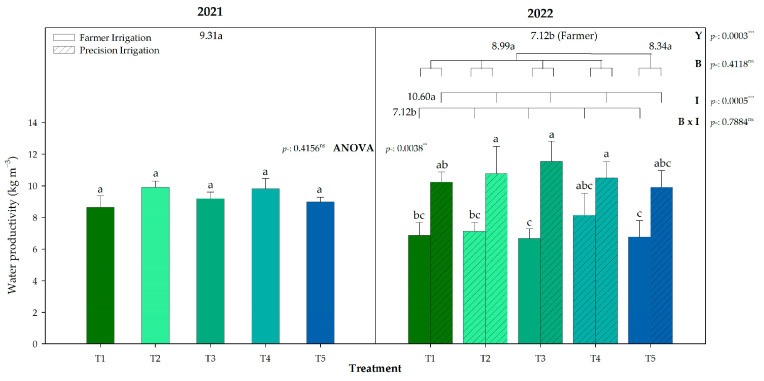
Irrigation water productivity (WP_I_) for biostimulation treatments under the different irrigation programs (farmer or precision irrigation) during the years 2021 and 2022. Bars represent means ± SE (*n* = 4). Average value for each factor (Y: year; B: biostimulation; I: irrigation) is shown. Different letters indicate significant differences according to Duncan’s test (*p* < 0.05). **: *p* < 0.01; ***: *p* < 0.001; *ns*: not significant.

Regarding the yield obtained, the irrigation water productivity (WP_I_) did not differ between treatments within the same irrigation program (Figure 5), averaging 9.31 kg m^−3^ in 2021. In 2022, as the yield decreased but the irrigation water did not, the water productivity also decreased to 7.12 kg m^−3^. Nevertheless, in 2022, the irrigation factor showed significant differences, increasing the WP_I_ by 3.5 kg per m^3^ in the PI treatments, as the water was reduced with respect to FI. In 2022, the treatments showed significant differences (*p* < 0.01), with T3 under PI having the highest WP_I_, whereas the T3 and T5 under FI had the lowest. It must be highlighted that the WP_I_ in T4 was not affected by the irrigation program, as it showed the highest values for farmer irrigation in 2022.

## 3. Discussion

The precision irrigation (PI) implemented in a table grape orchard led to water savings of 1336 m^3^ per ha, a reduction of 30% compared to the water management based on the farmer’s irrigation (FI) practices (Figure 1), which increased the irrigation water productivity (WP_I_) by 49% (Table 2).

The PI treatment was implemented through the use of soil water sensors that monitor the soil water storage at different depths. This technique has been used to assess irrigation strategies for grapevines in arid regions [23], with assessments every 5 days. In our trial, real-time monitoring of the soil volumetric water content was crucial to establish the threshold for reducing the irrigation in PI (Figure 2), in a similar way that has been used in other crops [8].

This water reduction was reflected in the physiological parameters of the crop, such as stem water potential (Ψ_S_), which has been proven to be a good indicator of the plant’s water status, as it is very sensitive to water deficit [24]. The Ψ_S_ values for FI for the entire trial period oscillated between −0.60 and −0.77 MPa (Table S1), coinciding with those reported by other authors [25,26] for well-irrigated vines. For the irrigation reduction treatment, PI, the Ψ_S_ decreased by 0.2 to 0.3 MPa compared to the FI treatment. The minimum Ψ_S_ value occurred at ripening, reaching −0.90 MPa. This value did not imply a decrease in yield (Table 2). Other authors found that above −1.2 MPa, the table grape yield was not affected [25,26,27]. In our trial, only the influence of the irrigation factor was observed in this parameter. The table grapes subjected to PI did not recover their optimal water status, even at 134 DAFBs, 9 days after being irrigated again under the FI program. Both the untreated vines (T5) and the ones treated with the different biostimulants (T1–T4) showed equivalent Ψ_S_ values. Similar findings were reported by Meggio et al. [28], in whose experiment Ψ_S_ was not influenced by the biostimulation with protein hydrolysates. This contrasts with other biostimulation trials, where after a water stress, Ψ_S_ was increased when biostimulants were applied, whether they were extract of algae, herbs, and plant oils in citrus [29], or seaweeds extracts in table grape [30].

Once the irrigation reduction in PI was implemented, the leaf gas exchange parameters, stomatal conductance (Lc) and photosynthesis (Pn), were reduced in PI compared to FI. Due to the high climatic demand and the low irrigation doses in PI, vines were forced to regulate the stomata so that the roots could absorb water from the soil [31].

Regarding biostimulation, seaweed extract biostimulants have been reported to improve the gas exchange parameters in *Vitis vinifera* L. after a water stress period [30]. Tombesi et al. [32] linked the stomatal sensitivity to vapor pressure deficit to *Ascophyllum nodosum* extract biostimulation, helping the vines better manage stress periods. Although in our trial only the treatments with foliar seaweed extract applied in 2021 showed an increase in Pn, this was not consistent over time. Nevertheless, the role of the root system in water absorption remained crucial.

Under water-stress conditions, the root system often adapts by increasing its depth and density to access deeper water reserves [33]. This adaptive mechanism ensures that the water absorbed by the roots can be efficiently transported to the leaves, maintaining photosynthesis and leaf conductance even under challenging conditions. In our trial, the fine root growth between both years was not significant, but the biostimulated plants under PI irrigation (B_PI_) showed a trend to grow, averaging 6.5 mg cm^−3^, while T5_FI_ did not (Figure 3A). This could be due to the combination of both factors, which have been separately reported to influence root growth. On the one hand, long-term deficit irrigation, either continuous (50% ET_c_ for the entire year) or regulated (60 to 25% ET_c_ for noncritical periods), has been shown to increase the root length density in apricot trees [33]. On the other hand, some biostimulants, such as biochar, have been shown to increase the fine roots biomass in 15-year-old vines, but this growth was associated with their radial growth, as they obtained higher diameters than the nontreated ones [34].

Apart from root growth, our results showed that the root mycorrhization rate increased between 2021 and 2022, from an average rate of 23% to 65% (Figure 3B). This increase was significant for all the biostimulated treatments under both irrigation programs, but not for T5, the nonbiostimulated treatment, whose colonization rate was only increased under reduced irrigation. This result contrasts with Schreiner et al. [35], who, after a deficit irrigation period in pre- or post-veraison, observed a decrease in the fine root growth, compensated by an increase in the arbuscular mycorrhiza colonization. Mycorrhizal fungi, particularly arbuscular mycorrhiza (AM), are naturally present in more than 85% of higher plant species [36]. They are, indeed, considered as biostimulants by themselves [20], and, among their effects against abiotic stress conditions, they can enhance the water uptake from dry soils [37], preventing cellular dehydration [38]. Some biostimulants have been shown to promote the root colonization by arbuscular mycorrhizal fungi [29,39]. However, this effect is not applicable to all biostimulant types or trials, as certain biostimulants such as biochar have been reported to not have an influence on the glomalin concentration, an indirect marker of mycorrhiza colonization [34]. Nevertheless, in 2022, the mycorrhization rate of the nonbiostimulated treatment showed a trend of increasing due to the irrigation reduction program (30% T5_FI_, 53% T5_PI_). This trend was not observed in the biostimulated treatments, as they already showed a higher colonization rate. As mycorrhizal fungi improve tolerance against abiotic stresses [40], nontreated plants can use this symbiosis as a mechanism to alleviate it.

In a mycorrhizal association, the host plant provides the AM fungi with organic carbon and lipids for their maintenance [41]. Related to the carbon transport, An et al. [42] found that the expression of a glucose transporter from the SWEET family is enhanced in mature arbuscular mycorrhiza in symbiosis with *Medicago truncatula*. Our results showed that the mycorrhization rate increased yearly in the biostimulated treatments compared to the untreated control (Figure 3B). The root starch content was also increased, but only under farmer irrigation conditions, primarily affecting the biostimulated treatments (Figure 4). In contrast, under the reduced irrigation program, both the untreated control and biostimulated treatments presented the same starch concentration. Considering that the host plant provides the AM fungi with organic carbon, mainly in the form of glucose, the roots from the biostimulated treatments, which had a higher mycorrhization rate, would have required the plant to allocate more sugars to support the fungi. This increased allocation would create a soluble sugars deficit in the biostimulated plants. To mitigate this, the plant might convert starch into sugars, which may explain why we did not observe any change in root soluble sugar concentration (Figure 4).

Several factors can influence the water and nutrient use efficiency, with the mycorrhiza colonization being one of them, although in our trial we did not observe a linear relationship between them (Figure 3B and Figure 5).

For the vines under FI, the yield in 2022 decreased by 10% compared to the previous year, reflecting the typical alternate bearing behavior observed in table grapes [43], although we did observe a mitigation of the yearly yield fluctuations linked to the irrigation management, as the yield obtained in PI was a 10% higher than the FI program the same year, increasing it by 3.55 t ha^−1^. Further research needs to be conducted to confirm if an irrigation reduction, carried out in a noncritical period such as post-veraison [31], can also have an influence in the alternate bearing.

Related to the grape yield, biostimulated treatments (T1–T4) increased (not significantly) the grape production in around 3 t ha^−1^. Among them, some treatments were more influenced by irrigation management than others, with treatment T3 being the one that most increased its yield under PI, and T4 being the only one that numerically decreased it. All treatments under the PI program increased their yield precocity; a significant bunch number ripened and were ready to harvest earlier than the treatments under FI. The combination of PI with biostimulation, particularly in T3 and T4, resulted in collecting nearly 90% of the total within the first three harvests, whereas the treatments under the farmer irrigation only achieved 58% of the total during the same period. Similar findings were observed by Pinillos et al. [26], that an earlier harvest was achieved by reducing the post-veraison irrigation by 75%.

Former research has shown that an irrigation water reduction of up to 40% during the post-veraison period in table grapes can be the threshold from which a decrease in grape production is observed; otherwise, the irrigation water productivity (WP_I_) is increased [27]. The water savings of 30% in PI treatments led to an increase of 49% in the irrigation water productivity (WP_I_), which was significant for the biostimulated treatments composed of seaweed extract (T2) or microorganisms (T3), and the untreated ones (T5). Peculiarly, the combination of seaweed extract and microorganisms (T4) did not significantly increase WP_I_ as this treatment had the highest under FI; therefore, its increase was not significant under PI. Similar results have been reached in grapevines, although deficit irrigation improves WP_I_ by itself, and once it is combined with a soil conditioner, the WP_I_ shows its greatest improvement [44].

The combined effect in table grapes of biostimulation and reduced water inputs in the post-veraison period, controlled by sensors, allows farmers to achieve yield precocity and significantly increased water productivity. The tested biostimulants acted by enhancing the mycorrhization rate, supplying the necessary sugars for its growth and promoting the root growth. Overall, this combination promotes more efficient and sustainable farming practices; therefore, we can determine that biostimulants have a high potential for promoting the fruit yield of grapevine in drought-prone regions.

## 4. Materials and Methods

### 4.1. Experimental Conditions

This trial was carried out in a commercial vineyard located in Alhama de Murcia, Region de Murcia, Spain (37°45′33″ N, 1°19′46″ W), during the years 2021 and 2022. The experimental plot of table grapes (*Vitis vinifera* L.) cv. Sweet celebration comprised a cultivated area of 3675 m^2^, with 300 adults vines in a 3.5 m × 3.5 m planting frame on a clay soil (48% clay, 24% silt, 28% sand).

The vines were covered by a net and were irrigated through a drip irrigation system, established by a line with 4 drippers per vine with a nominal flowrate of 3.8 L h^−1^. The crop evapotranspiration (ET_c_) was calculated according to the FAO method [45], by multiplying the crop coefficient (K_c_) with the reference evapotranspiration (ET_0_). The K_c_ values used in this trial have been previously calculated for the area [46]. For the different phenological stages: preflowering, flowering and fruit set, berries’ development, berries’ growth, veraison, ripening and harvest, and late harvest, the K_c_ values were 0.22, 0.30, 0.35, 0.45, 0.45, 0.45, and 0.40, respectively [46]. The amount of water applied for each treatment was controlled via volumetric water meters and the fertilization was the same for both irrigation programs.

The climate in this region is categorized as dry Mediterranean type, belonging to Köppen “Bsh” classification [47], with high temperatures in the summer, soft winters, and rare precipitations averaging 282.6 mm per year for the last 10 years [48]. Daily reference evapotranspiration (ET_0_), rainfall, vapor pressure deficit (VPD), and temperatures were obtained from the agroclimatic station “AL-41” located in La Calavera (Alhama de Murcia) belonging to the “Murcia Agrometeorological Information Service” network [46].

### 4.2. Experimental Design

A randomized trial design was established in plots corresponding to three adjacent rows of five vines. The experimental unit was the three central vines located in the middle row, while the other two served as borders. In both years of study (2021 and 2022), five treatments were evaluated with four replicates (*n* = 4) based on the type of biostimulant applied and a control without application, which were applied via fertigation at three phenological stages: (i) sprouting, at 61 and 40 days prior to flowering for 2021 and 2022, respectively; (ii) full bloom; and (iii) between the fruit set and pea-sized berries, at 48 and 22 days after full bloom (DAFBs) for 2021 and 2022, respectively. The treatments corresponded to those described in Table 3.

Furthermore, to determine the effect of the use of biostimulants for agronomic management, their incorporation as a factor was analyzed at two levels: biostimulant application (T1–T4) vs. no application (T5) in both years of study. Additionally, in 2022, the plots were randomly divided into two subplots to evaluate irrigation scheduling as a factor at two levels: farmer irrigation (FI) and precision irrigation (PI). In the vines irrigated as FI, irrigation was scheduled according to the farmer’s traditional criteria, satisfying around 115% of the ET_c_. On the other hand, the irrigation criterion for the vines under PI was based on soil water depletion, allowing the soil water content to decrease by 10% of its field capacity. The soil water content was monitored in real time using soil water status sensors. Differential irrigation for PI started at 49 DAFBs (5 July 2022) and was equal to FI at 125 DAFBs (19 September 2022).

The composition of the biostimulant products is as follows: Amalgerol^®^ is formulated with seaweeds and vegetable extracts, essential oils, distillate of paraffin oil, and 21% total organic carbon. Seamac Rhizo^®^ is a combination of seaweed extract (*Ascophyllum nodosum*, 15%) with vegetable extracts, amino acids, and nutritional elements. Accudo^®^ is a plant-growth-promoting rhizobacteria, *Bacillus paralicheniformis* (RTI-184, 26 g L^−1^ [49]).

The pest and disease management was carried out by attending to the commercial program equally for all the treatments. Among the farmer treatments for all plots, the yearly application of a foliar seaweed extract (*Ecklonia* spp.) and a mycorrhizal inoculant (*Glomus iranicum*, MycoUp 360^®^, Symborg) at fruit setting must be highlighted.

### 4.3. Field Measurements

#### 4.3.1. Crop Phenology

Crop phenology was monitored using the agrometeorological index of growing degree days (GDDs) [50] according to Equation (1):

GDDs = [(T_max_ + T_min_)/2)] − T_Base_, (1)

where T_max_ and T_min_ are daily maximum and minimum air temperature, respectively, and T_Base_ is the base temperature for table grape growth, T_Base_ = 10 °C [51]. The GDDs accumulation was calculated from sprouting (−62 days after full bloom, DAFBs).

#### 4.3.2. Soil Volumetric Water Content

The irrigation factor was controlled through an FDR probe model Drill & Drop (Sentek Technologies, Stepney, Australia) that was installed in three replicates within each irrigation program, in the wet bulb 10 cm apart from the dripper. These probes measure the soil volumetric water content every 10 cm between 10 and 60 cm depth. Measurements were obtained minutely and averaged every 10 min. Data were registered in a datalogger and uploaded to the Irriman platform [52]. Data obtained were normalized to the field capacity at each depth (θ_FC_; m^3^ m^−3^).

#### 4.3.3. Vines Water Status

Foliar gas exchange parameters, such as stomatal conductance (Lc) and net photosynthesis (Pn), were measured with a portable photosynthesis system, LI-COR 6800 (LI-COR INC., Lincoln, NE, USA). The established CO_2_ concentration was 400 μmol mol^−1^, and the photosynthetic photon flux density was 1200 µmol mol^−2^ s^−1^; the temperature and relative humidity corresponded to the environment. The measurements were taken monthly at solar midday, in 2 mature leaves of the central vines for each replicate (*n* = 8 per treatment).

Stem water potential (Ψ_s_) was measured monthly at the solar midday from the start of the differential irrigation (49 DAFBs) in 2022, using a pressure chamber type Scholander model Pump-Up (PMS Instrument Company, Albany, OR, USA) on 1 mature leaf per replicate (*n* = 4 per treatment). The leaves were covered with foil bags 2 h prior to the measurement.

#### 4.3.4. Root Evaluations

After harvest, 160 DAFBs in 2021 and 134 DAFBs in 2022, root density was calculated by extracting an unaltered core cylinder (5 × 5 cm) of samples per replicate, which were extracted 5 cm away from the vines’ nearest dripper. Fine roots with a diameter lower than 1 mm were washed and dried at 60 °C until reaching a constant weight.

From those roots, the starch and soluble sugar were extracted and determined according to the Somogyi–Nelson method [53,54]. The reaction was measured at 520 nm using glucose as standard in a UV–visible spectrophotometer (model UV-1650 PC, Shimadzu, Kyoto, Japan).

A similar core sample per replicate was extracted to assess root mycorrhizal colonization. Roots were washed with distilled water and stained with parker ink and vinegar, following a modified protocol from Phillips and Hayman [55]. To determine the mycorrhizal colonization, root sections (*n* = 100) were assessed using an optical microscope (Olympus CX43, Olympus, Tokyo, Japan) and classified as positive or negative mycorrhizal structures, calculating the percentage of mycorrhization according to the method of McGonigle et al. [56].

### 4.4. Harvest and Irrigation Water Productivity

Fruit yield (t ha^−1^) was assessed in the three central vines of each plot. The harvest dates were based on marketable quality standard; therefore, they were distributed among 6 to 7 different dates, normally taking place between early June and late September (I: 6 August 2021, 29 July 2022; II: 10 August 2021, 16 August 2022; III: 17 August 2021, 22 August 2022; IV: 26 August 2021, 30 August 2022; V: 7 September 2021, 20 September 2022; VI: 20 September 2021, 30 September 2022; VII: 30 September 2021). All harvests were recorded to assess yield precocity. In addition to the yield data, the bunch number and bunch weight in the individual harvests were recorded.

Irrigation water productivity (WP_I_) was determined as kg of fruit per m^3^ applied [57,58].

### 4.5. Statistical Analysis

The described variables were analyzed using linear mixed models (LMMs) that included the effect of biostimulant treatments or factors and their interactions (biostimulant use, irrigation criterion, and year) in the fixed part of the model and the plots as the random part of the model (*p* < 0.05). In 2022, a hierarchical structure was added to the model, including subplots as random effects nested within the original plots. Prior to the analyses, assumptions were tested: the normality of the error distributions of each dependent variable was evaluated according to the Shapiro–Wilk test (*p* < 0.05), and the homoscedasticity of the variances was evaluated with the Levene test (*p* < 0.05), using absolute residuals to minimize the possible effect of outliers and improve the power of the test [59,60,61]. When significant differences by treatments or factors were detected, means were separated using Duncan’s test (*p* < 0.05). All the statistical analyses were carried out using the InfoStat software version 2020e and its interface with R [62].

## Figures and Tables

**Table 1:** Average gas exchange parameters, net photosynthesis (Pn) and stomatal conductance (Lc), and midday stem water potential (Ψ_s_) for the years 2021 (−9 to 76 DAFBs) and 2022 (−11 to 134 DAFBs) (individual dates can be found in Table S1) according to the different treatments (T) or the biostimulation (B), irrigation (I), and year (Y) factors.

Year (Y)	Treatment (T)	Biostimulation (B)	Irrigation (I)	Pn (µmol m^−^² s^−^¹)		Lc (mol m^−^² s^−^¹)		Ψ_s_ (MPa)
2021	T1	Yes	Farmer	10.28	a	0.1161	a	
	T2			9.87	a	0.1039	ab	
	T3			8.85	ab	0.0978	ab	
	T4			8.53	ab	0.0871	b	
	T5	No		7.80	b	0.0814	b	
	*ANOVA*			*0.0132* *		*0.0283* *		
2022	T1	Yes	Farmer	11.84		0.1550		
	T2			12.66		0.1716		
	T3			12.22		0.1527		
	T4			13.07		0.1733		
	T5	No		13.18		0.1616		
	*ANOVA*			*0.3738 ^ns^*		*0.4626 ^ns^*		
			Y	*<0.0001* ***		*<0.0001* ***		
			T	*0.2852 ^ns^*		*0.6914 ^ns^*		
			Y × T	*0.0387* *		*0.3967 ^ns^*		
2022 Season		Yes	Farmer	8.82		0.1234		−0.66
From 49 DAFBs on			Precision	7.54		0.0915		−0.89
		No	Farmer	9.25		0.117		−0.65
			Precision	7.12		0.0881		−0.82
			B	*0.9844 ^ns^*		*0.4857 ^ns^*		*0.1323 ^ns^*
			I	*0.0015* **		*0.0008* ***		*<0.0001* ***
			B × I	*0.3290 ^ns^*		*0.8245 ^ns^*		*0.2336 ^ns^*

Means, *n* = 4. Different letters indicate significant differences between treatments according to Duncan’s test (*p* < 0.05) for that parameter and date. *p*-values for ANOVA are shown in *italics*. *: *p* < 0.05; **: *p* < 0.01; ***: *p* < 0.001; *ns*: not significant.

**Table 2:** Total yield and yield from the harvest I to III or IV to VII (or VIII in 2021) for the biostimulation treatments under different irrigation programs (farmer and precision irrigation) during the years 2021 and 2022.

Year	Irrigation	Treatment	Total Yield	Yield I to III	Yield IV to VII
(Y)	(I)	(T)	t ha^−^¹	t ha^−^¹	t ha^−^¹
2021	Farmer	T1	38.12	18.41	19.71
		T2	43.67	21.45	22.22
		T3	40.56	24.21	16.35
		T4	43.28	22.46	20.82
		T5	39.63	18.03	21.60
		*ANOVA*	*0.3165 ^ns^*	*0.4096 ^ns^*	*0.6193 ^ns^*
2022	Farmer	T1	35.67	21.76	13.91
		T2	37.02	20.72	16.31
		T3	34.67	12.85	21.82
		T4	42.32	27.54	14.78
		T5	35.15	24.24	10.91
		*ANOVA*	*0.7943 ^ns^*	*0.3050 ^ns^*	*0.4215 ^ns^*
2022	Precision	T1	39.44	33.97	5.47
		T2	41.49	34.08	7.42
		T3	44.51	40.53	3.98
		T4	40.50	39.53	0.97
		T5	38.19	31.43	6.76
		*ANOVA*	*0.8941 ^ns^*	*0.4139 ^ns^*	*0.5153 ^ns^*
	Y		*0.0485* *	*0.8200 ^ns^*	*0.0398* *
	T		*0.3074 ^ns^*	*0.6713 ^ns^*	*0.9465 ^ns^*
	Y × T		*0.8945 ^ns^*	*0.1002 ^ns^*	*0.2141 ^ns^*
2022	B		*0.6573 ^ns^*	*0.4811 ^ns^*	*0.1361 ^ns^*
	I		*0.2125 ^ns^*	*<0.0001* ***	*<0.0001* ***
	B × I		*0.8870 ^ns^*	*0.1995 ^ns^*	*0.1409 ^ns^*

*: *p* < 0.05; ***: *p* < 0.001; *ns*: not significant.

**Table 3:** Biostimulation treatments applied via fertigation at different phenological stages of the vine during 2021 and 2022.

Treatment	Sprouting	Full Bloom	Fruit Set to Pea-Sized Berries
	L ha^−1^		
T1 ^A^: Amalgerol^®^	10	5	5
T2 ^A^: Seamac Rhizo^®^	5	5	5
T3 ^A^: Accudo^®^	1	1	1
T4: Seamac Rhizo^®^ + Accudo^®^	4 1	4 1	4 1
T5: Control	-	-	-

^A^ During the preconditioning year (2021), treatments T1, T2, and T3 were applied together with another seaweed extract biostimulant, Seamac PCT^®^ (*Ascophyllum nodosum*, 15%), applied via foliar spray at 0.30% at 44 days before bloom, and 12 and 20 days after full bloom.

## Data Availability

Data will be made available on request.

## Supplementary Materials

The following supporting information can be downloaded at: https://www.mdpi.com/article/10.3390/plants13233424/s1, Table S1: Gas exchange parameters, net photosynthesis (Pn) and stomatal conductance (Lc), and midday stem water potential (Ψs) for the different phenological stages in 2021 and 2022, according to the different treatments or the biostimulation (B), irrigation (I), and year (Y) factors.
