# Supplementary material for: Combined Effects of Deficit Irrigation and Biostimulation on Water Productivity in Table Grapes
Source: Plants (Basel). 2024 Dec 6;13(23):3424. doi: 10.3390/plants13233424 (PMC11644483; doi:10.3390/plants13233424)
Supplement: Supplementary file 1 [file plants-13-03424-s001.zip › plants-3302976-supplementary.pdf]

Table S1. Gas exchange parameters, net photosynthesis (Pn) and stomatal conductance (Lc), and midday stem water potential (Ψ_s) for the different phenological stages in 2021 and 2022, according to the different treatments or the biostimulation (B), irrigation (I) and year (Y) factors.

Year (Y)	Phenological stage	Treatment	Biostimulation (B)	Irrigation (I)	Pn (μmol m^{-2} s^{-1})		Lc (mol m^{-2} s^{-1})	Ψ_s (MPa)
2021	I2: Flowering	T1	Yes	Farmer	17.15	a	0.2251	
	BBCH 64	T2			16.66	a	0.2142	
	−9 DAFB	T3			15.21	ab	0.1924	
	230 °C GDD	T4			14.41	b	0.1605	
		T5	No		14.44	b	0.1768	
		ANOVA			*0.0335**		*0.3078*^{ns}	
	J: Fruit set	T1	Yes	Farmer	6.93		0.0505	
	BBCH 71	T2			8.35		0.035	
	28 DAFB	T3			7.53		0.0328	
	590 °C GDD	T4			5.97		0.037	
		T5	No		5.45		0.0257	
		ANOVA			*0.4655*^{ns}		*0.0874*^{ns}	
	M1: Beginning veraison	T1	Yes	Farmer	7.55		0.0664	
	BBCH 83	T2			6.76		0.0588	
	62 DAFB	T3			5.31		0.051	
	1076 °C GDD	T4			6.58		0.0563	
		T5	No		4.73		0.0396	
		ANOVA			*0.7997*^{ns}		*0.8372*^{ns}	
	N: Ripening	T1	Yes	Farmer	9.49		0.1224	
	BBCH 85	T2			7.7		0.1077	
	76 DAFB	T3			7.37		0.115	
	1296 °C GDD	T4			7.17		0.0946	
		T5	No		6.58		0.0834	
		ANOVA			*0.8398*^{ns}		*0.8584*^{ns}	
2022	I2: Flowering	T1-T4	Yes	Farmer	14.76		0.191	
	BBCH 60							
	−11 DAFB	T5	No		15.93		0.190	
	190 °C GDD			B	*0.3718*^{ns}		*0.9738*^{ns}	
	L: Cluster closure	T1-T4	Yes	Farmer	11.73		0.135	
	BBCH 79							
	30 DAFB	T5	No		12.5		0.150	
	665 °C GDD			*B*	*0.6706*^{ns}		*0.5957*^{ns}	
	M1: Beginning veraison	T1-T4	Yes	Farmer	12.14		0.176	-0.60
	BBCH 83			Precision	11.51		0.154	-0.86
	63 DAFB	T5	No	Farmer	14.92		0.223	-0.60
	1168 °C GDD			Precision	10.97		0.173	-0.72
				B	*0.4779*^{ns}		*0.2139*^{ns}	*0.1822*^{ns}
				I	*0.1594*^{ns}		*0.1763*^{ns}	*0.0056***
				B x I	*0.2967*^{ns}		*0.5685*^{ns}	*0.2245*^{ns}
	N: Ripening	T1-T4	Yes	Farmer	11.15		0.151	-0.61
	BBCH 89			Precision	7.6		0.077	-0.89
	98 DAFB	T5	No	Farmer	9.38		0.084	-0.59
	1771 °C GDD			Precision	7.13		0.062	-0.88

			B	*0.3288*^{ns}	*0.0060***	*0.7408*^{ns}
			I	*0.0214**	*0.0022***	*<0.0001****
			B x I	*0.5674*^{ns}	*0.0571*^{ns}	*0.7131*^{ns}
N: Ripening	T1-T4	Yes	Farmer	3.16	0.043	-0.77
BBCH 89			Precision	3.49	0.043	-0.93
134 DAFB	T5	No	Farmer	3.44	0.044	-0.78
2305 °C GDD			Precision	3.26	0.030	-0.87
			B	*0.9738*^{ns}	*0.3993*^{ns}	*0.1889*^{ns}
			I	*0.9191*^{ns}	*0.3196*^{ns}	*0.0001****
			B x I	*0.7362*^{ns}	*0.3329*^{ns}	*0.1156*^{ns}

Means, n = 4. Different letters indicate significant differences between treatments according to Duncan's test (p<0.05) for that parameter and date. p-values for ANOVA are shown in *italics* *: p<0.05; **: p<0.01; ***: p<0.001; ns: not significant.
